# A scalable approach to the computation of invariant measures for high-dimensional Markovian systems

**DOI:** 10.1038/s41598-018-19863-4

**Published:** 2018-01-29

**Authors:** Susanne Gerber, Simon Olsson, Frank Noé, Illia Horenko

**Affiliations:** 1 Johannes-Gutenberg University of Mainz, Faculty of Biology, Staudinger Weg 9, 55128 Mainz, Germany; 2 Center of Computational Sciences Mainz (CSM), Staudinger Weg 9, 55128 Mainz, Germany; 3 Freie Universität Berlin, Department of Mathematics and Computer Science, Arnimallee 6, 14195 Berlin, Germany; 4 Università della Svizzera Italiana, Faculty of Informatics, ICS, Via G. Buffi 13, TI-6900 Lugano, Switzerland

## Abstract

The Markovian invariant measure is a central concept in many disciplines. Conventional numerical techniques for data-driven computation of invariant measures rely on estimation and further numerical processing of a transition matrix. Here we show how the quality of data-driven estimation of a transition matrix crucially depends on the validity of the statistical independence assumption for transition probabilities. Moreover, the cost of the invariant measure computation in general scales cubically with the dimension - and is usually unfeasible for realistic high-dimensional systems. We introduce a method relaxing the independence assumption of transition probabilities that scales quadratically in situations with latent variables. Applications of the method are illustrated on the Lorenz-63 system and for the molecular dynamics (MD) simulation data of the *α*-synuclein protein. We demonstrate how the conventional methodologies do not provide good estimates of the invariant measure based upon the available *α*-synuclein MD data. Applying the introduced approach to these MD data we detect two robust meta-stable states of *α*-synuclein and a linear transition between them, involving transient formation of secondary structure, qualitatively consistent with previous purely experimental reports.

## Introduction

Gaining knowledge from data is critically dependent upon our ability to prune-out biases originating from the data acquisition and data analysis procedures, as well as distinguishing these biases from the inherent properties of the underlying system. For example, computation of simple data ensemble averages - such as the empirical expectation or the empirical probability density function (p.d.f.) - can provide results that depend very strongly on the data sampling procedure and measurement settings. Such results would not allow a direct assessment of the intrinsic *invariant* characteristics of the underlying dynamical system that produced these measurements. In many areas of research, invariant Markov measure (or an invariant Markov distribution) is recognised as the key object allowing for a robust analysis of the data by providing a canonical view into the dynamics of underlying systems^1–5^. For example, in network science and computational sociology, the Markovian invariant measure gives a ranking of the nodes in underlying graphs, as in the Google PageRank algorithm, for example^6^. In biophysical molecular dynamics (MD) the invariant measure provides equilibrium probabilities for different molecular conformations^7–10^ and is used in computations of various dynamical characteristics, including the most probable transition pathways^11^ and in comparison with experimental data^12,13^. In fluid mechanics and in climate research the Markovian invariant measure enables the identification of the coherent flow structures^14^ and aids understanding the change of an equilibrium system’s response to the external perturbations^15,16^.

Conventional computational methods used for the practical inference of the invariant measures rely on *Ulam’s approach*^17^. The first step of this approach involves the creation of a finite-dimensional, discretised representation of the system’s phase space with a fixed finite number *n* of boxes/compartments {*x*(1), *x*(2), …, *x*(*n*)}. The second step involves the computation of the discretised time series of measurement data in this representation: {*X*(0), *X*(*τ*), …, *X*(*s*), …, *X*(*S*)}, where *τ* is a time discretisation step. For every time *s* going from 0 to *S*, every *X*(*s*) takes one and only one of the *n* discrete values {*x*(1), *x*(2), …, *x*(*n*)}. This time series is assumed to be Markovian: in order to obtain *X*(*s* + *τ*) for every *s* it is necessary and sufficient to know only the value of *X*(*s*). This time series is used to compute the *transfer operator* - a square (*n* × *n*)-matrix of conditional probabilities ${\boldsymbol{\Lambda }}=\{{{\rm{\Lambda }}}_{ij}\}={\mathbb{P}}[X(s+\tau )=x(i)|X(s)=x(j)]$ for all *s*. Defining the column vector of probabilities as $\pi (s)=\{{\mathbb{P}}[X(s)=x\mathrm{(1)]},\ldots ,{\mathbb{P}}[X(s)=x(n)]\}$ and making use of the *law of total probability*^10,18^, one can write an exact equation describing the time-evolution of *π*(*s*) as:

1 $$\pi (s+\tau )={\boldsymbol{\Lambda }}\pi (s\mathrm{).}$$

This is referred to as the master equation of a Markov process and its fixed point *μ*

2 $$\mu =\mathop{\mathrm{lim}}\limits_{N\to \infty }{{\boldsymbol{\Lambda }}}^{N}\pi \mathrm{(0),}$$

is called an *invariant measure* (or *invariant distribution*) of the Markov process. In the third and final step of *Ulam’s approach*, the invariant measure is calculated as the eigenvector correspondent to the eigenvalue λ = 1 of the transfer operator **Λ** - i.e., as the non-negative solution of the system of linear equations *μ* = **Λ***μ* and ${\sum }_{i=1}^{n}{\mu }_{i}=1$.

In practical applications the transfer operator **Λ** is either assumed to be explicitly known and given (e.g., as in the case of the PageRank algorithm^6^) - or it is estimated from the discretised data, for example by maximising the observational log-likelihood function

3 $$ {\mathcal L} ({\boldsymbol{\Lambda }})=\sum _{i,j}^{n}{N}_{ij}{\mathrm{log}{\rm{\Lambda }}}_{ij}\to \mathop{{\rm{\max }}}\limits_{{{\rm{\Lambda }}}_{ij}},$$

where ${N}_{ij}={\sum }_{s=0}^{S-\tau }\chi (X(s+\tau )={x}_{i})\chi (X(s)={x}_{j})$ (with *χ* being an indicator function) contains the numbers of observed transitions between *x*(*j*) and *x*(*i*) in the data, after a step *τ*. Problem (3) can be solved analytically, resulting in the widely used *empirical frequency estimator*:

4 $${{\rm{\Lambda }}}_{ij}=\frac{{N}_{ij}}{\sum _{j}^{n}{N}_{ij}}\mathrm{.}$$

Validity of the log-likelihood function formulation (3) - as well as the validity of the resulting maximum log-likelihood *empirical frequency estimator* (4) - rest heavily on the validity of the implicit assumption about the independence of transitions between different states. This independence assumption allows us to write down the likelihood as a product of probabilities and the respective log-likelihood in (3) as a sum of log-likelihoods, resulting in a very simple analytical solution (4). However, this assumption can be easily violated in realistic systems when different transition probabilities jointly depend on some latent variables or processes. In such a case applying the *empirical frequency estimator* (4) when computing the invariant measure *μ* would introduce a bias, deforming the results and interpretations. This problem is exacerbated by the absence of practical computational tools that can assess the validity of this independence assumption.

The second bottleneck of the empirical frequency estimation (4) for **Λ** is induced by the intrinsic uncertainty of this estimate, growing polynomially in *n* for a fixed statistics size *S*/*τ*^10,19^. Practical manifestation of this bottleneck is the so-called overfitting phenomenon: i.e., when *S*/*τ* is small and *n*(*n*−1)/2 is large, then there is not enough data to have a reliable statistics of transitions *N*_*ij*_, meaning that the *empirical frequency estimator* (4) will fit the training data well, but generalizes poorly and fails to reproduce validation data.

The third main bottleneck of the popular invariant measure computation procedures is the sheer numerical cost of computing the *μ* from a given transition operator **Λ**. If **Λ** does not exhibit any particular structure (i.e., if it is not sparse), the overall numerical cost of the invariant measure computation scales as ${\mathscr{O}}({n}^{3})$, thereby confining its practical applicability to relatively small systems (i.e., *n* cannot routinely exceed 10,000 or 20,000 when working on commodity hardware)^8–10^. For the applications in network science and computational social sciences some successful examples for *n* being of the order 10^9^–10^10^ have been shown^6^. However, in these particular cases a very strong sparsity of **Λ** was used, a sparsity that is usually not given a priori for many realistic applications in MD and in the geosciences^19^.

Summarising, in the case of the data-driven analysis the three main bottlenecks of standard procedures based on Ulam’s approach are: (i) biasing independence assumptions involved in a practical computation of the transfer operator **Λ**; (ii) overfitting phenomenon; and (iii) the numerical cost scaling for computing *μ* from unstructured **Λ** with a large *n*. In the following we will present a simple idea allowing to construct a joint algorithmic remedy for these bottlenecks.

## Latent Markovian inference of the invariant measure

**Figure 1:**
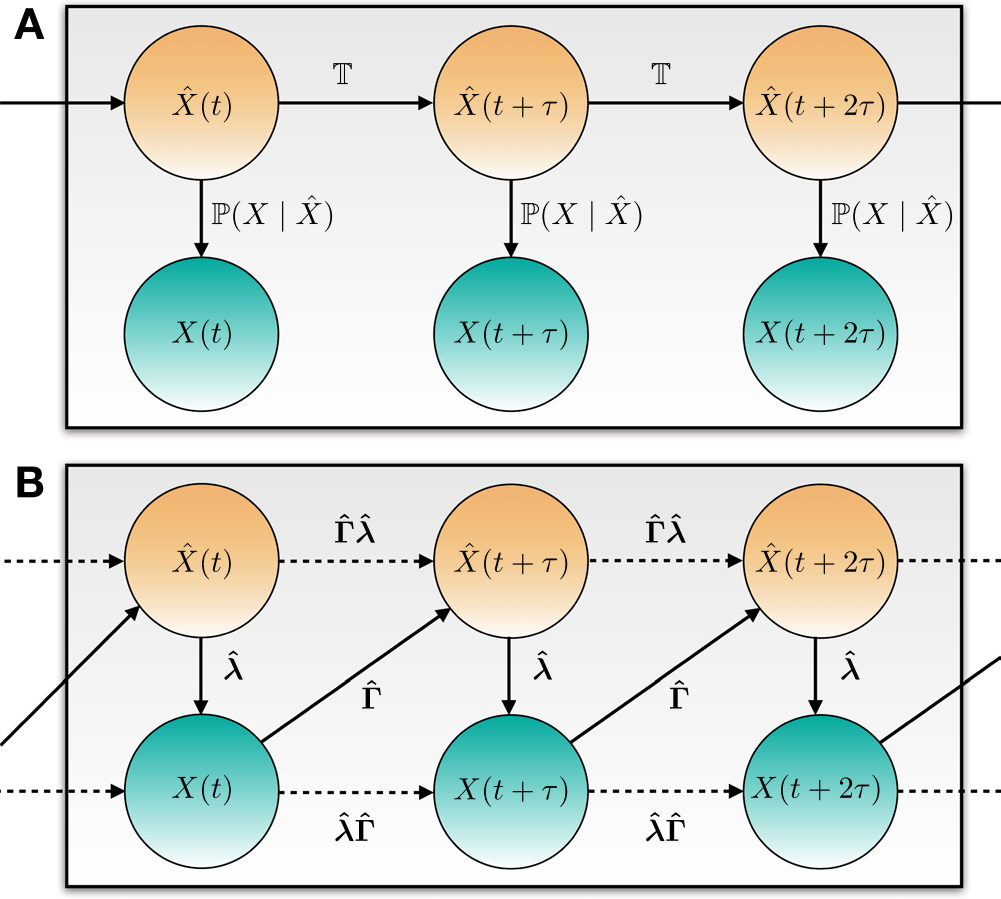
A Bayesian net illustration of the Hidden Markov Models (**A**) and the latent Markov models (**B**). The conditional relations are shown as directed edges and the random variables as coloured edges (observable variables are cyan and the latent/hidden variables are orange). Dashed edges represent the derived transfer operators in the full and latent space.

Let $\{\hat{X}\mathrm{(0),}\,\hat{X}(\tau ),\ldots ,\,\hat{X}(S)\}$ be a latent (i.e., unobserved) categorical process that is defined on a statistically disjoint set of (yet unknown) categories $\{\hat{x}\mathrm{(1)},\hat{x}\mathrm{(2)},\ldots ,\hat{x}(K)\}$ (with *K* < *n*). Deploying the law of total probability, we can establish the Bayesian relation between $\hat{X}(s+\tau )$ and *X*(*s*), and between *X*(*s*) and $\hat{X}(s)$. The former relation is achieved for all *s* through the conditional probabilities ${\hat{{\boldsymbol{\Gamma }}}}_{kj}={\mathbb{P}}[\hat{X}(s+\tau )=\hat{x}(k)|X(s)=x(j)]$ and the latter through the conditional probabilities ${\hat{{\boldsymbol{\lambda }}}}_{ik}={\mathbb{P}}[X(s)=x(i)|\hat{X}(s)=\hat{x}(k)]$. Subsequently, it is straightforward to validate that an optimal probability-preserving reduced approximation of the full relation model (1) takes a form:

5 $$\pi (s+\tau )=\hat{{\boldsymbol{\lambda }}}\hat{{\boldsymbol{\Gamma }}}\pi (s),$$

where $\{{\pi }_{i}(s)\}={\mathbb{P}}[X(s)=x(i)]$, $i=\mathrm{1,}\ldots ,n$. $\hat{{\boldsymbol{\Gamma }}}$ is a rectangular (*K* × *n*) column-stochastic matrix (i.e., sums of the elements in each column equal to one and all of the matrix elements are non-negative) whereas $\hat{{\boldsymbol{\lambda }}}$ is a rectangular (*n* × *K*) matrix that is also column-stochastic (Fig. 1). Formal derivation of the formula (5) and the proof of a probability preservation property of (5) can be found in the supplement. In being essentially a matrix-vector formulation of the law of total probability, the reduced model (5) is exact in the Bayesian sense and involves no further approximations.

For a given discretised times series of the observational data $\{X\mathrm{(0)},X(\tau ),\ldots ,X(S)\}$, both of the unknown matrices $\hat{{\boldsymbol{\lambda }}}$ and $\hat{{\boldsymbol{\Gamma }}}$ can be computed iteratively - e.g., deploying the log-likelihood maximisation (as described above for the case of the standard master equation (1)). Practically, for this purpose one can use the algorithms like PLSA (Probabilistic Latent Semantic Analysis - an expectation maximisation algorithm^20,21^) or DBMR (direct Bayesian model reduction - a clustering algorithm^19^). For further details related to a comparison of these two alternative methods we refer to ref.^19^. As was demonstrated in ref.^19^, estimation of $\hat{{\boldsymbol{\lambda }}}$ and $\hat{{\boldsymbol{\Gamma }}}$ with DBMR scales much more favourably with problem dimension both in terms of the space and time complexity. Following the same line of argument for the latent Markov model representation (5), one obtains that the overall computational cost of the DBMR algorithm until reaching the convergence when applied to (5) will scale as ${\mathscr{O}}(K\,{\rm{\min }}({n}^{2},S/\tau ))$ and will require no more than ${\mathscr{O}}({\rm{\min }}({n}^{2},S/\tau ))$ of memory. In order to avoid the problem of overfitting it is important to guarantee that the overall number of free parameters in the latent Markov model (5) does not become too large compared with the size *S*/*τ* of the available data statistics - and does not exceed the number of free parameters in the original master equation without latent variables (1), resulting in a simple algebraic expression for the upper bound of *K*: $K < \frac{{n}^{2}}{\mathrm{(2}n-\mathrm{1)}}$.

The key idea that will help us to adopt this latent Markov model (5) for the computation of invariant measures in the presence of latent process $\{\hat{X}\mathrm{(0),}\,\hat{X}(\tau ),\ldots ,\,\hat{X}(S)\}$ is based on the following simple observation: inserting (5) into (2) and keeping *K* fixed we obtain

6 $$\mu =\mathop{\mathrm{lim}}\limits_{N\to \infty }{(\hat{{\boldsymbol{\lambda }}}\hat{{\boldsymbol{\Gamma }}})}^{N}\pi \mathrm{(0)}=\hat{{\boldsymbol{\lambda }}}\mathop{\mathrm{lim}}\limits_{N\to \infty }{({\hat{{\boldsymbol{P}}}}_{K})}^{N-1}\hat{{\boldsymbol{\Gamma }}}\pi \mathrm{(0),}$$

where ${\hat{{\boldsymbol{P}}}}_{K}=\hat{{\boldsymbol{\Gamma }}}\hat{{\boldsymbol{\lambda }}}$ is a (*K* × *K*) column-stochastic *reduced transfer operator*. If there exists a unique dominant eigenvector ${\hat{\mu }}_{K}$ correspondent to the eigenvalue 1.0 of the matrix ${\hat{{\boldsymbol{P}}}}_{K}$ (i.e., ${\hat{\mu }}_{K}={\hat{{\boldsymbol{P}}}}_{{\bf{K}}}{\hat{\mu }}_{K}$), then we can express *μ* in terms of ${\hat{{\boldsymbol{P}}}}_{K}$ and $\hat{{\boldsymbol{\lambda }}}$ using the Perron-Frobenius theorem^18^:

7 $$\mu =\frac{1}{\sum _{k=1}^{K}{\hat{\mu }}_{k}}\hat{{\boldsymbol{\lambda }}}{\hat{\mu }}_{K}\mathrm{.}$$

It is very straightforward to validate that setting K = 1 transforms (5) into a memoryless Bernoulli model with *μ* becoming the empirical probability density function (p.d.f.) of the data. Setting *K* > 1 results in the memory-one Markovian models - where the memory is carried by the latent process $\hat{X}$ with K states. Finally, it is very straightforward to validate that for K = n and $\hat{X}(t)\equiv X(t)$ we get that the latent model (5) becomes equivalent to the common Markov model (2). In another words, changing K one can “scramble” through the whole range of models - scaling from memoryless Bernoulli (*K* = 1), over latent Markov (1 < *K* < *n*) to a full Markov (*K* = *n*) model.

The overall cost of computing *μ* from (7) consists of the cost for computing $\hat{{\boldsymbol{\lambda }}}$ and $\hat{{\boldsymbol{\Gamma }}}$ (being ${\mathscr{O}}(K\,\min ({n}^{2},S/\tau ))$ for DBRM algorithm^19^), cost for computing ${{\boldsymbol{P}}}_{K}$ (being ${\mathscr{O}}(K(K-\mathrm{1)}{n}^{2})$), cost for computing the dominant eigenvector ${\hat{\mu }}_{K}$ for ${\hat{{\boldsymbol{P}}}}_{K}$ (being ${\mathscr{O}}({K}^{3})$) and cost for computing *μ* from $\hat{\lambda }$ and ${\hat{\mu }}_{K}$ in (7) (being ${\mathscr{O}}({K}^{2}n)$). Adding all terms together and keeping only the leading-order terms in *n* and *K* we obtain that the overall numerical cost for computing the *n*-dimensional invariant measure *μ* in a presence of the *K*-dimensional latent (unobserved) process $\hat{X}$ is ${\mathscr{O}}(K(K-\mathrm{1)}{n}^{2}+K\,{\rm{\min }}({n}^{2},S/\tau )+{K}^{3})$. Analogously, the overall memory consumption of this method in the leading order will be no more then ${\mathscr{O}}({\rm{\min }}({n}^{2},S/\tau )+{K}^{3})$. These results mean that in the situations where the latent dimension *K* < *n* is fixed and independent of the observed dimension *n*, the cost and the memory consumption for this latent Markovian invariant measure computation will both scale quadratically in *n* - resulting in a more favourable cost scaling than the standard computation based on the full transfer operator **Λ** in (1) (that scales cubically in *n* for general unstructured and non-sparse matrices **Λ**).

Please note that the latent Markov models (LMMs) deployed above are not the same thing as the hidden Markov models (HMMs). LMMs are a particular Markovian case of the latent Bayesian models (LBMs)^19–21^. Hidden Markov Models (HMMs) and Latent Markov Models (LMMs) are very different in the way how the conditional relations between the observed and the hidden/latent variables are established. In Fig. 1 one can see the graphical representation of these model relations. On the mathematical side, the main implication of this difference is the fact that - in context of LMMs - one can establish a direct relation between the consecutive realizations *X*_*t*−1_ and *X*_*t*_ of the observed process. This can be done in a straightforward manner by adopting the reduced master equation (5) that is inferred directly from the data - i.e., we circumvent the explicit inference of the latent space realization. In contrast, HMMs would require the explicit inference of the hidden process $\{\ldots ,{\hat{X}}_{t-1},{\hat{X}}_{t},{\hat{X}}_{t+1},\ldots \}$ and of the conditional probability tensor ${\mathbb{P}}[{\hat{X}}_{t}={\hat{x}}_{k}|{X}_{t-1}={x}_{i},{X}_{t}={x}_{j}]$ - and would not allow obtaining the reduced master equation (5) directly from the data. On the computational side, as a result of these differences, the scaling of a memory complexity in LMM inference as ${\mathscr{O}}(K(n-\mathrm{1)}+n+min(S/\tau ,{n}^{2}))$ is favourable compared to that of HMMs which grows as ${\mathscr{O}}(KS/\tau +K{n}^{2})$^19^, where *S*/*τ* is the sample size of data statistics and *n* the data dimension. This in turn allows LMMs to scale better to a big data setting.

Next, we are going to address the question of selecting the optimal *K* - as well as deciding whether the latent Markov computation (7) or the standard Markov computation based on the *empirical frequency estimator* (4) without the latent processes provide a better description of the discretized data series $\{X\mathrm{(0),}\,X(\tau ),\ldots ,\,X(S)\}$. Many of the standard model selection tools from the area of machine learning can be used to answer this question, in the following examples we will use two of them: (i) a cross-validation approach and (ii) the Bayesian or Akaike information criteria. Cross-validation involves pooling the data into two parts - the training and validation sets. Candidate models are fitted to the training set and the model exhibiting the best performance on the validation data set is selected as the most adequate. However, in many situations (especially when the underlying model is non-stationary or when the available data is very sparse) this approach can lack robustness since it will crucially rely on the way how the original data is separated into the training and the validation sets. The family of information criteria, e.g., Akaike Information Criterion (AIC), Bayesian Information Criterion (BIC), and others^22^ provide an alternative which does not rely on ad hoc sub-division of the data prior to validation. These approaches measure and compare the posterior parameter uncertainty of different models - allowing to perform this comparison by means of the very simple formula involving the model quality/performance (measured in terms of the optimal log-likelihood value) and penalising the model complexity (measured as the overall number of free model parameters). Then, a model with the minimal value of the information criterion provides the most adequate and the most statistically-significant among the considered models (in terms of the posterior model probability) description for a given data^22^. When applying information criteria one needs to keep in mind that they can only give an asymptotical comparison between the models - i.e., they must be used with caution when the statistics size *S*/*τ* is small^22^. Combination of the estimator formula (7) with these procedures of *K*-selection allows a fully-automated inference of infarinat measures that is not relying on any user-defined tuning parameter. In the following we will illustrate this automated approach on two examples.

## Application Examples

### Lorenz-63 oscillator: quantification of the bias, scalability tests

**Figure 2:**
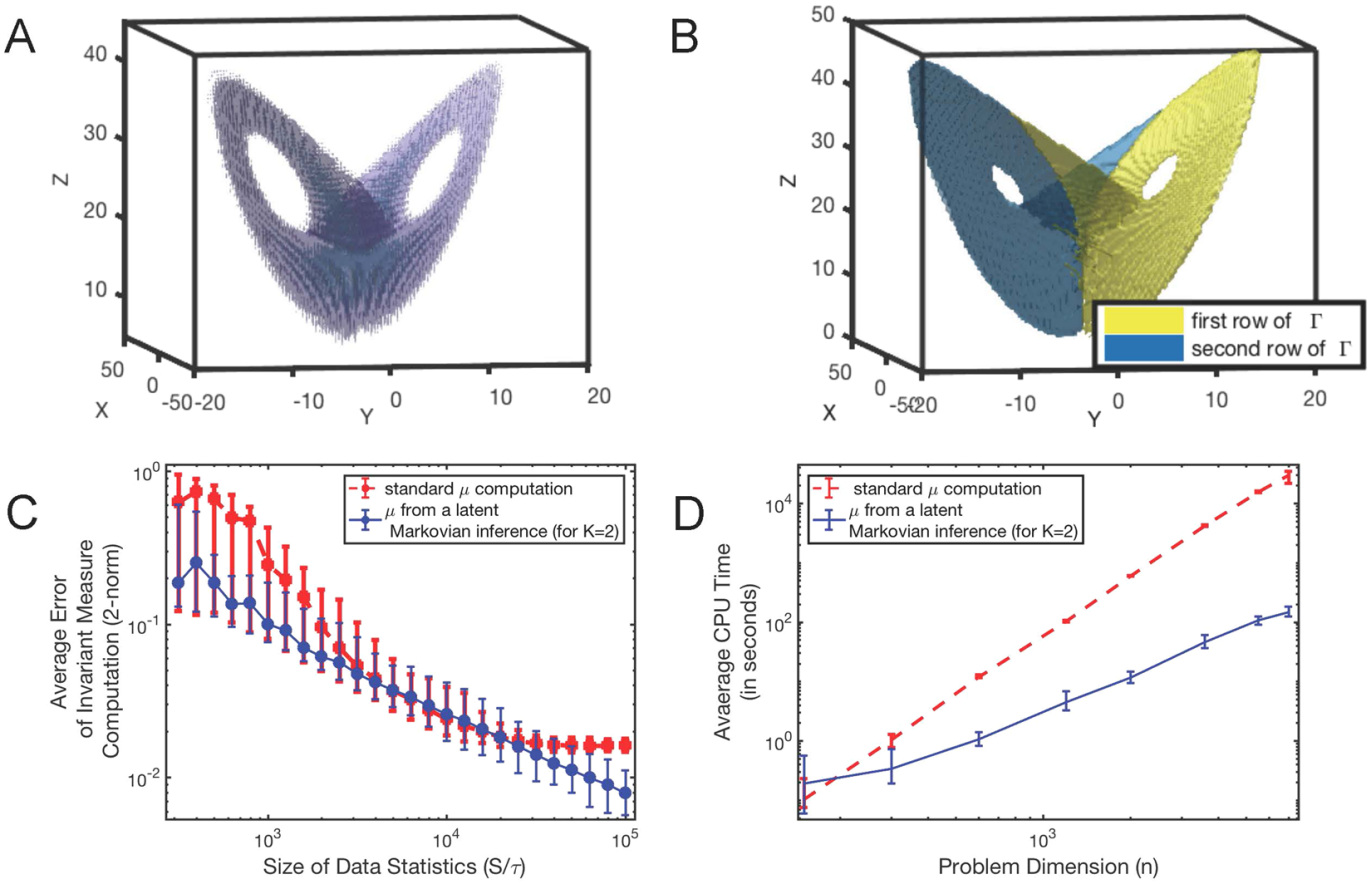
Application of the latent Markov invariant measure computation (5, 6) to the data from a Lorenz-oscillator simulation and comparison with a standard Ulam’s procedure based on (1, 4): (**A**) resulting invariant measure *μ*; (**B**) affiliation of the original discretisation boxes to the identified latent states; (**C**) comparison of the error statistics as a function of the statistics size *S*; and (**D**) comparison of the computational costs as a function of problem dimension *n*.

Lorenz-63 is a deterministic dynamical system often used as a benchmark^2,3,23,24^. We use the standard setting of Lorenz model parameters (*σ* = 10, *ρ* = 28, *β *= 8/3) and generate the observational data with a time step *τ* = 10^−3^ and an overall simulation time *S* = 10^3^ by means of the adaptive Runge-Kutta-method. In the first step of Ulam’s approach, these data are discretised on a uniform 100 × 100 × 100 box grid, resulting in a discretised time series $\{X\mathrm{(1),}\,X\mathrm{(2),}\ldots ,\,X(S)\}$ describing the jumps between *n* = 10^6^ of the 3D boxes. Next, we compute the standard invariant measure *μ* as the normalised dominant eigenvector of the sparse *empirical frequency matrix*. Since this matrix is sparse we use a specialised Lanzcos-Krylov-solver as implemented in Matlab (command *eigs*() in MATLAB versions 7 and later). Then we compute *μ* from the latent Markov model (5, 6) with the latent dimension *K* = 2 (in the left upper panel of Fig. 2). Comparison both by means of the model cross-validation and by means of the information criteria (AIC and BIC)^22^ shows that the invariant measure based on the latent Markov model provides a significantly better description of the data. It is very instructive to inspect the corresponding matrix $\hat{{\rm{\Gamma }}}$ of the conditional probabilities, relating the observed process *X*(*s*) and the latent process $\hat{X}(s+\mathrm{1)}$. This matrix is obtained with the DBMR algorithm^19^ (MATLAB implementation available at https://github.com/SusanneGerber), its row *k* contains element zero in a position *j* if there is no conditional relation between the observed state *x*(*j*) (i.e., some particular 3D box in our case) and the latent state $\hat{x}(k)$ - and contains element one if there is such a relation. As can be seen from the upper right panel of Fig. 2, for *K* = 2 the optimal latent Markov model (5) decomposes the original states/boxes in two latent sets: latent state $\hat{x}\mathrm{(1)}$ - with conditional probability 1.0 containing all of the original boxes/compartments on the right wing of the Lorenz-attractor (marked yellow), and the latent state $\hat{x}\mathrm{(2)}$ - with conditional probability 1.0 containing the original boxes/compartments on the left wing of the Lorenz-attractor (marked blue). It means that the identified latent process $\{\hat{X}\mathrm{(0),}\,\hat{X}(\tau ),\ldots ,\,\hat{X}(S)\}$ is a process switching between the two wings of the Lorenz-attractor and that the transitional probabilities between the boxes are not all independent (as implied by the *empirical frequency estimator* (4)) but “belong together” and are conditionally dependent on the wing (left or right).

Lower panels of the Fig. 2 show a comparison of the statistics of errors (lower left) and statistics of computational costs for the standard method (red lines) and for the latent Markov computation of the invariant measure (blue). Every error bar indicates average values and their 95% confidence intervals obtained from the ensembles of 500 independent Lorenz data trajectories. For the standard *μ* computation we use the efficient sparse Lanzcos-Krylov solver to obtain the dominant eigenvectors of (4). As can be seen from the two plots, latent Markov computation outperforms a standard computation based on (1, 4), enabling quantification and separation of the biases coming from the overfitting and from the implicit independence assumption, respectively (Fig. 2).

### Analysis of α-Synuclein MD data

The protein *α*-synuclein is abundant in the brain and has been associated with a number of neurodegenerative conditions including Morbus Parkinson. Despite of the apparent importance of *α*-synuclein indicated in many studies, the scope of biological functions and it’s exact role in disease development remains poorly understood. Some experimental evidence suggest that aggregation of *α*-synuclein monomers has a cytotoxic effect and may trigger the neurodegenerative process in brain cells^25^. Further, single-molecule experiments tie this agglomeration process to a large variety of metastable conformational states of the *α*-synuclein monomers^26,27^. However, gaining a more detailed understanding of these processes (e.g., by means of MD simulations) is hampered by a combination of two factors that make the MD simulations of *α*-synuclein difficult: (i) its a fairly large disordered protein (140 amino acid residues) which makes it necessary to use very large solvation boxes which makes reaching experimentally relevant time-scales difficult and (ii) many state-of-the-art molecular mechanics forcefields do not describe disordered proteins well. Moreover, even if MD simulation of such a system becomes feasible, the intrinsic complex properties of the data as well as the relatively short sampling time spans may prohibit application of the conventional Markov modelling machinery. Overcoming this would enable the quantitative extraction of information about thermodynamics and molecular kinetics of conformational transitions of the system. However, as of yet we are limited to the most basic statistical quantities such as the empirical averages and p.d.f.^28^.

To evaluate whether the latent Markov model approach allows us to overcome problems we face when using conventional Markov modelling tools, we turned to a previously published 11 *μ*s trajectory, conducted in the Amber12 forcefield with the TIP4P-D water-model which has been shown to agree well with experimental data^28^. Molecular features were extracted from all frames of the trajectory: including sines and cosines of all backbone torsions, as well as the minimum C-*α* distances between all pairs of secondary structure elements. The secondary structure elements involve the following residue ranges; [3; 11], [17; 19], [21; 32], [41; 44], [45; 47], [52; 55], [66; 68], [70; 78], [80; 83], [88; 89], [110; 113], [120; 122], [124; 126] and [133; 136], as defined in Uniprot entry P37840^29^. In total, this yields 647 features. These features were projected into a three dimensional space by means of Time-lagged Independent Component Analysis (TICA, lag time 50 nanoseconds)^30,31^, this space was subsequently clustered into 200 disjoint states using K-means clustering. MD data processing and analysis was performed using MDTraj and PyEMMA^32,33^.

Inspection of the obtained discrete time series $\{X\mathrm{(1),}\,X\mathrm{(2),}\ldots ,\,X(S)\}$ reveals that it arrives in a set of terminal states which are not reversibly connected to the other Markov states. This is a very common practical problem emerging in Markov model analysis of limited MD data. The immediate practical implication of this is that the corresponding empirical Markov model estimate (1, 4) is irreversible, leading to a spurious concentration of probability mass at this terminal state (Figure S1). A number of constrained estimators have been proposed to ensure a reversible Markov model is obtained from such data^34–36^, yet these do not guarantee meaningful results - and can introduce an additional bias. In this example we note that the use of a reversible estimator in fact exacerbates this problem (Figures S1 and S2). Consequently, we can conclude that the use of standard Markov tools for these data is not directly possible and we are indeed limited to determining basic statistical quantities such as the empirical averages and p.d.f. However, as explained above, empirical averages and p.d.f. are not invariant with respect to sampling and do not allow an insight into intrinsic dynamical properties of the molecule that could be otherwise accessible through the Markovian invariant measure.

**Figure 3:**
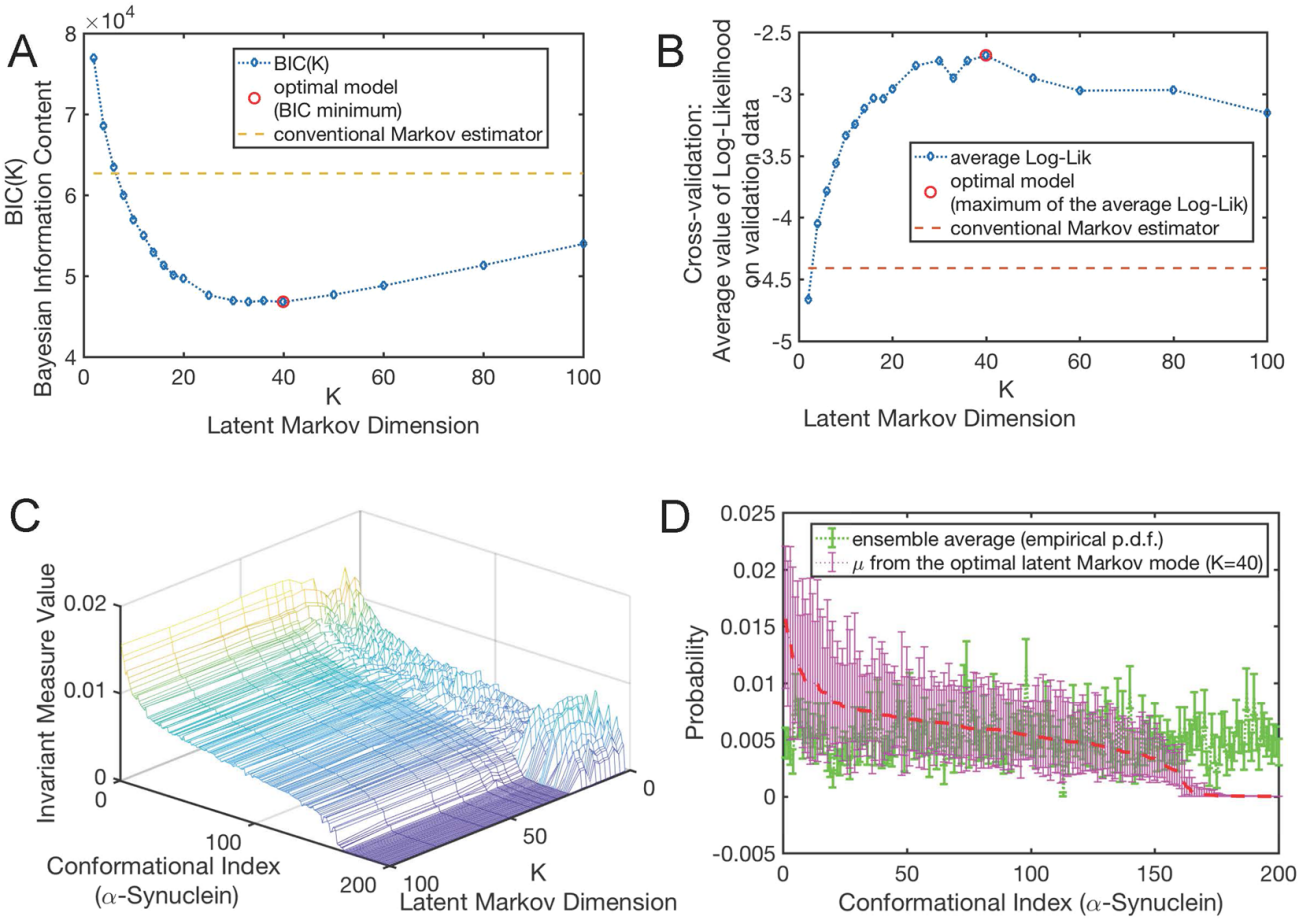
Analysis results for the α-Synuclein MD data: (**A**) choice of the optimal latent dimension *K* by means of the Bayesian Information Criterion; (**B**) choice of the optimal latent dimension *K* by means of the cross-validation procedure; (**C**) invariant measure *μ* as a function of latent Markov dimension *K*; (**D**) comparison of the empirical p.d.f. and the invariant measure *μ* obtained from (5, 6).

Next, we computed latent Markov models for different *K* values between *K* = 1 (corresponding to the memoryless Bernoulli case with *μ* becoming the empirical p.d.f.) and *K* = 100 (that is beyond the “overfitting” range of *K* = *n*^2^/(2*n−*1) ≈ 100). We selected the optimal *K* by both cross-validation and using the Bayesian Information Criterion^37^ - and comparing to the values obtained for the standard Markov model (1, 4) (Fig. 3A,B). This comparison reveals that the latent models provide a significantly better description of these data, and the optimal latent dimension of *K* = 40 (Fig. 3A,B). The invariant measure (*μ*) of the latent Markov model converges to a stable estimate already at *K* ≈ 20, (see Fig. 3C). For *K* = 40 a comparison of the invariant measure of the latent Markov model with the empirical p.d.f. is shown with 95%-confidence intervals computed by a standard non-parametric bootstrap sampling^38^.

**Figure 4:**
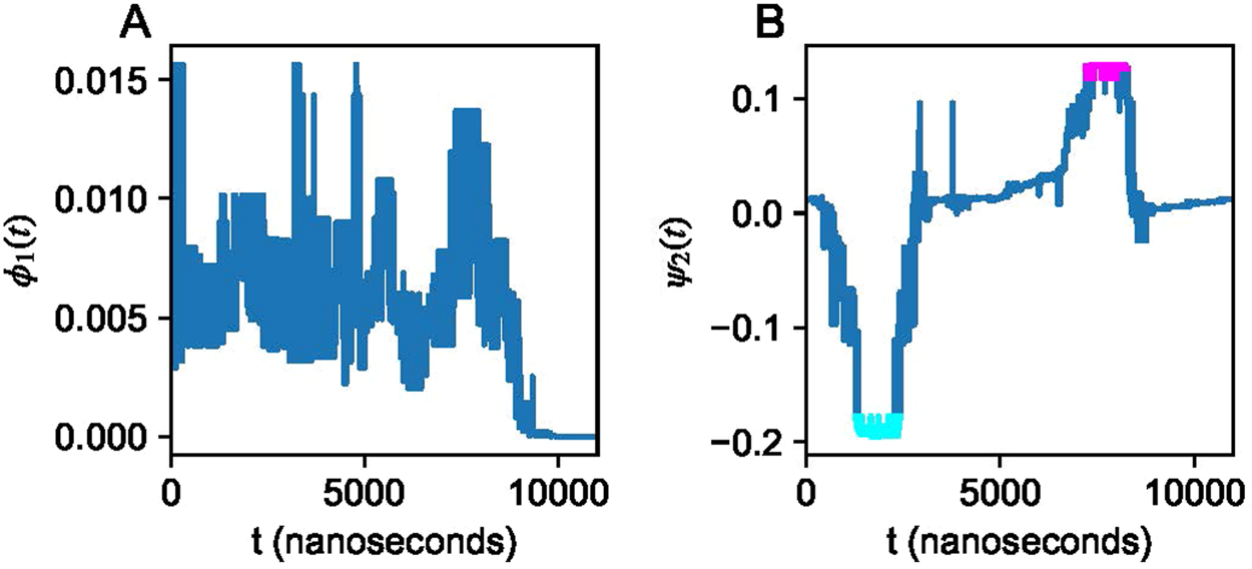
Discrete time series of α-synuclein projected onto (**A**) the leading invariant measure *ϕ*_1_ = *μ* and (**B**) the second invariant measure normalized by the first invariant measure *Ψ*_2_. Meta-stable states 1 and 2 are highlighted with magenta and cyan colors in B.

The optimal latent Markov model (*K* = 40) was chosen for further analysis. Interestingly, we find that the non-reversibly connected states get assigned a vanishing probability (Fig. 4A), which alleviates the distortion observed in the invariant measure (Fig. S1) with the standard empirical estimator (4). For molecular systems, the second (and higher) invariant measure(s) describes the probability flow in conformational space associated with the slow, finite relaxation time-scales of the Markov model, and is often used to identify meta-stable configurations^39^. The empirical estimator, both with and without reversibility constraint, attributes the slowest process exchange with the non-reversibly connected states (Fig. S2). The corresponding measure for the latent Markov model however identifies three reversibly connected meta-stable configurations (Fig. 4B). For further analysis below, we annotate the two most temporally distant of these states, highlighted by cyan and magenta colors, as states 1 and 2, respectively.

**Figure 5:**
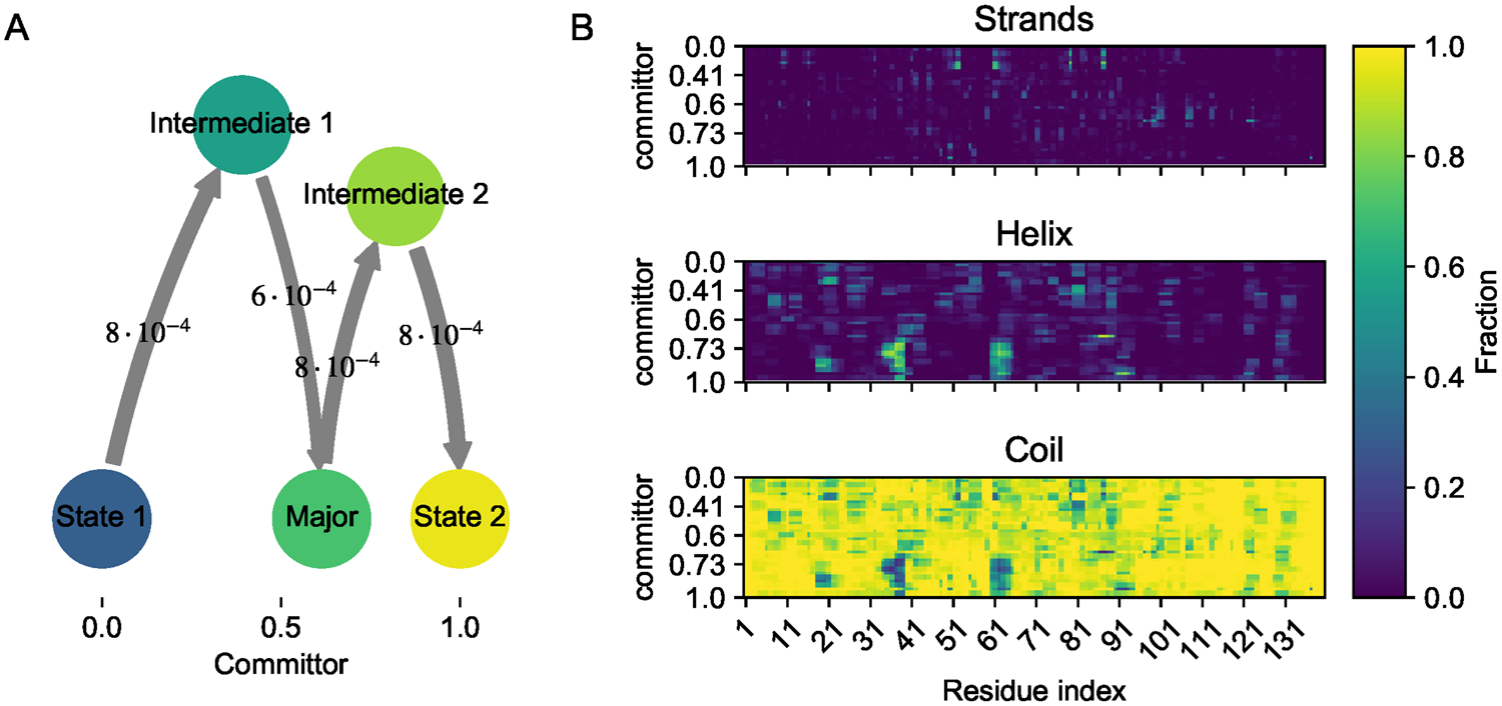
Net-flux along the committor from state 1 to state 2 (**A**), changes in relative protein secondary structure (Helix, Coil and Strands) for each residue position along the committor from states 1 to 2 (**B**).

To better understand the conformational changes happening in between states 1 and 2 we perform a coarse-grained transition path analysis^11,40^. This analysis allows us to compute committor probabilities, which here is the probability of arriving in state 2 before returning to state 1. The Markov states were lumped together according to their committor probability, and a resulting network plot of the net-flux reveals a linear transition mechanism (Fig. 5A). Next, we projected the fraction of different secondary structure elements as a function of sequence onto the committor probability (Fig. 5B). While *α*-synuclein remains largely disordered with the majority of the residues not forming persistent secondary or tertiary structural arrangements, we observe that two meta-stable states 1 and 2 show some preferences for particular secondary structure in distinct regions of the primary sequence. State 1 adopts strand like conformation in residues surrounding position 53, 61, 80 and 88 - whereas state 2 adopts helical secondary configurations in residues around position 20, 37, 63 and 88. These regions coincide with secondary structures observed in experiments, including *β*-sheets observed in a solid-state NMR structure of an *α*-synuclein fibril^41^ and *α*-helical conformations observed in *α*-synuclein fragments bound to maltose binding protein^42^. The intermediate state 2 also shows similar helical structures in particular around residues 37 and 63. These results suggests that a slow conformational exchange process (with mean first passage time of around ~1–10 *μs*) in *α*-synuclein involves changing secondary structure propensities between strand-like conformations to helix like conformations. These changes are most prominent in residues surrounding position 53, 61, 80 and 88.

## Discussion

As demonstrated above, sparse experimental data, implicit mathematical assumptions (e.g., about the a priori independence of different transitions in the system) and computational issues (like the problem of overfitting and the computational cost scaling) may seriously bias or even prohibit the computation of the invariant measures with common tools, also for very simple and well understood dynamical systems like the Lorenz-oscillator. Deploying the algorithmic components from the Bayesian models with latent variables^19–21^ we construct a method allowing to compute the invariant measures from latent Markov models. As implied by the computational scaling results from Fig. 2D in comparison with common tools, this automated method can help to push the computational limits to much larger systems in the situations when the dimension of the latent process is smaller than the dimension of the observed process (Fig. 2C,D). And, as demonstrated above, this computation can be performed beyond the restrictive independence assumption required by the standard tools.

A general problem for any method dealing with data is induced by a necessity to distinguish between “what is in the data?” (i.e., what are the artefacts of data sampling/acquisition procedures, impact of statistics size, impact of dimension etc.) and “what is in the system?” (i.e., what are the true system’s characteristics and what are the biases introduced by the method). As discussed above, because of various reasons this distinction is particularly problematic in the MD data analysis. When analysing such data one only gains insight into these particular data - but not into the true underlying system per se. But understanding what the data really says is valuable, as it allows for a systematic assessment of the data, devoid of confounders introduced by the analysis method. As demonstrated for *α*-synuclein data, applying the common methods would not allow any additional insights that go beyond the very simple p.d.f. computations - whereas application of the latent Markov model estimation reveals a clear transition process (Fig. 4) with particular conformational characteristics (Fig. 5). Applying this methodology to molecular dynamics data enabled an analysis unattainable with standard Markov methods. In particular, since the simulation data involved absorbing states, invariant measures computed from Markov models obtained with conventional estimators were distorted by this property. To our surprise, we found the latent Markov model method to be insensitive to this deficiency in the data. While it remains unclear whether such favourable behaviour is to be expected in general for this approach, this is an intriguing finding with potential for more efficient data use in Markov modelling of molecular dynamics simulation data.

We expect this new tool for data-driven computation of invariant measures to be useful in many other application areas including network science, drug design and climate research. As discussed above, in all of these areas invariant measure computations are based on the same Ulam’s procedure and share the same limitations. An open source implementations of the algorithms presented herein is available online for download at https://github.com/SusanneGerber.

## Electronic supplementary material

Supplementary Information
