# Supplementary material for: A scalable approach to the computation of invariant measures for high-dimensional Markovian systems
Source: Sci Rep. 2018 Jan 29;8:1796. doi: 10.1038/s41598-018-19863-4 (PMC5789124; doi:10.1038/s41598-018-19863-4)
Supplement: Supplementary file 1 — Supplementary Information [file 41598_2018_19863_MOESM1_ESM.pdf]

Supplement for the paper: ”A scalable approach to the computation of invariant measures for high-dimensional Markovian systems”

Susanne Gerber[1,†], Simon Olsson[2,†], Frank Noé[2], Illia Horenko[3*]

[1] Johannes-Gutenberg University of Mainz, Faculty of Biology, Staudinger Weg 9, 55128 Mainz, Germany.

[2] Freie Universität Berlin, Department of Mathematics and Computer Science, Arnimallee 6, 14195 Berlin, Germany.

[3]Università della Svizzera Italiana, Faculty of Informatics, Via G. Buffi 13, TI-6900 Lugano, Switzerland

[*]To whom correspondence should be addressed; E-mail: horenkoi@usi.ch

[†]These authors contributed equally.

September 3, 2017

Derivation of the Latent Markov Model

Let $X = \{X(0), X(\tau), X(2\tau), \dots, X(s), \dots, X(S)\}$ be a discretised representation of the original system's dynamics on a fixed finite number n of boxes/compartments $\{x(1), x(2), \dots, x(n)\}$ in a phase space. The boxes are disjoint - meaning that for every $s \in [0, S]$ discretised process can take one and only one of the possible values from $\{x(1), x(2), \dots, x(n)\}$. Time discretisation step τ is supposed to be constant and fixed. The discrete time-series X is assumed to be Markovian: in order to obtain $X(s+\tau)$ for every s it is necessary and sufficient to know only the value of $X(s)$ - and not any other realisations $X(t), t < s$.

We shall be looking for a categorical process $\{\hat{X}(0), \hat{X}(\tau), \dots, \hat{X}(S)\}$ (being a reduced representation of the full categorical process X) that is defined on a reduced set of categories $\{\hat{x}(1), \hat{x}(2), \dots, \hat{x}(K)\}$ with $K < n$. Defining the column vector of probabilities as $\pi(s) = \{\mathbb{P}[X(s) = x(1), \dots, \ \mathbb{P}[X(s) = x(n)]\}$

and deploying the law of the total probability we can establish an exact Bayesian relation between the probability density $\hat{\pi}_{\hat{X}}(s+\tau)$ of this - still unknown - process and the full probability density of the observed process $\pi(s)$:

$$\hat{\pi}_{\hat{X}}(s+\tau) \;=\; \hat{\mathbf{\Gamma}}\pi(s), \tag{1}$$

where $\hat{\pi}_{\hat{X}}(s+\tau) = \left\{\mathbb{P}[\hat{X}(s) = \hat{x}(1), \ldots,\ \mathbb{P}[\hat{X}(s) = \hat{x}(K)]\right\}$ and $\hat{\mathbf{\Gamma}}_{kj} = \mathbb{P}\left[\hat{X}(s+\tau) = \hat{x}(k)|X(s) = x(j)\right]$ is the $K\times n$ matrix of Bayesian conditional probabilities relating the two processes $X(s)$ and $\hat{X}(s+\tau)$. This matrix can also be understood as a discrete probabilistic projection operator, playing in the following a similar role as the linear projection operators built of the dominant eigenvectors of the relation matrices in standard reduction methods (e.g., projection matrices built from the dominant eigenvectors of the data covariance matrix deployed in the Principal Component Analysis method or the Frobenius-eigenvectors of propagator and generator matrices used in the spectral reduction theory of Markov chains). The main conceptual difference of the $\hat{\mathbf{\Gamma}}$ matrix from the projection operators obtained in the standard reduction methods is that it is preserving the l_1-norm - thereby guaranteeing that the reduced process density $\pi_{\hat{X}}$ will always preserve the probability (i.e., $\sum_{i=1}^{K}\left\{\hat{\pi}_{\hat{X}}(s)\right\}_i \equiv 1$ and $\left\{\hat{\pi}_{\hat{X}}(s)\right\}_i \geq 0$ for all i). In contrast, the projection matrices in standard approaches like Markov spectral reduction theory and PCA are l_2 objects and do not automatically preserve a probability of the reduced density in this sense.

Next, we deploy a law of total probability to establish an exact Bayesian relation between the reduced (and unobserved) process $\hat{X}(s)$ and the observed process $X(s)$:

$$\pi(s) \;=\; \hat{\boldsymbol{\lambda}}\pi_{\hat{X}}(s), \tag{2}$$

where $\hat{\boldsymbol{\lambda}}_{ik} = \mathbb{P}\left[Y(s) = y(i)|\hat{X}(s) = \hat{x}(k)\right]$ is the $n \times K$ matrix of conditional probabilities connecting the two processes. Substituting (1) into (2) we obtain the latent Markovian model (equation 5 from the main manuscript):

$$\pi(s+\tau) \;=\; \hat{\boldsymbol{\lambda}}\hat{\mathbf{\Gamma}}\pi(s), \tag{3}$$

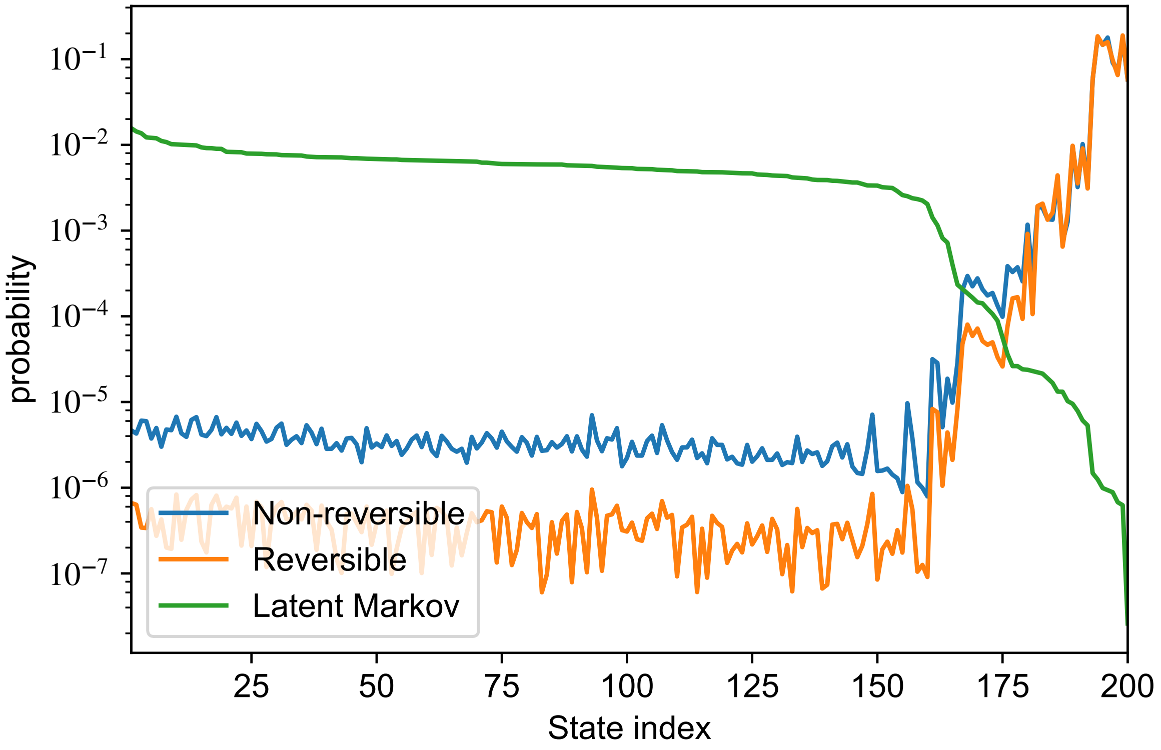

Figure S1: Comparisons of invariant measures computed for the 200 states of α-synuclein. Estimators MSM (reversible, non-reversible) and Latent Markov model. Lag-time 21 nanoseconds for all. Please also see the Fig. 3A and 3B from the main manuscript for the comparison of the corresponding model quality measures.

that now connects the observed processes not directly but rather indirectly - through a latent reduced process $\hat{X}$ that is defined on a categorical space of a smaller dimension K.

Since for every $k = 1, \ldots, K$ it is guaranteed that $\sum_{i=1}^{n} \hat{\boldsymbol{\lambda}}_{ik} \equiv 1$ and for every $j = 1, \ldots, n$ it is true that $\sum_{k=1}^{K} \hat{\boldsymbol{\Gamma}}_{kj} \equiv 1$ (preservation of conditional probability), it is straightforward to validate that if $\sum_{i=1}^{n} \pi_i(s) = 1$ then also $\sum_{i=1}^{n} \pi_i(s+\tau) = 1$, i.e., latent Markovian models (3) will always be probability-preserving.

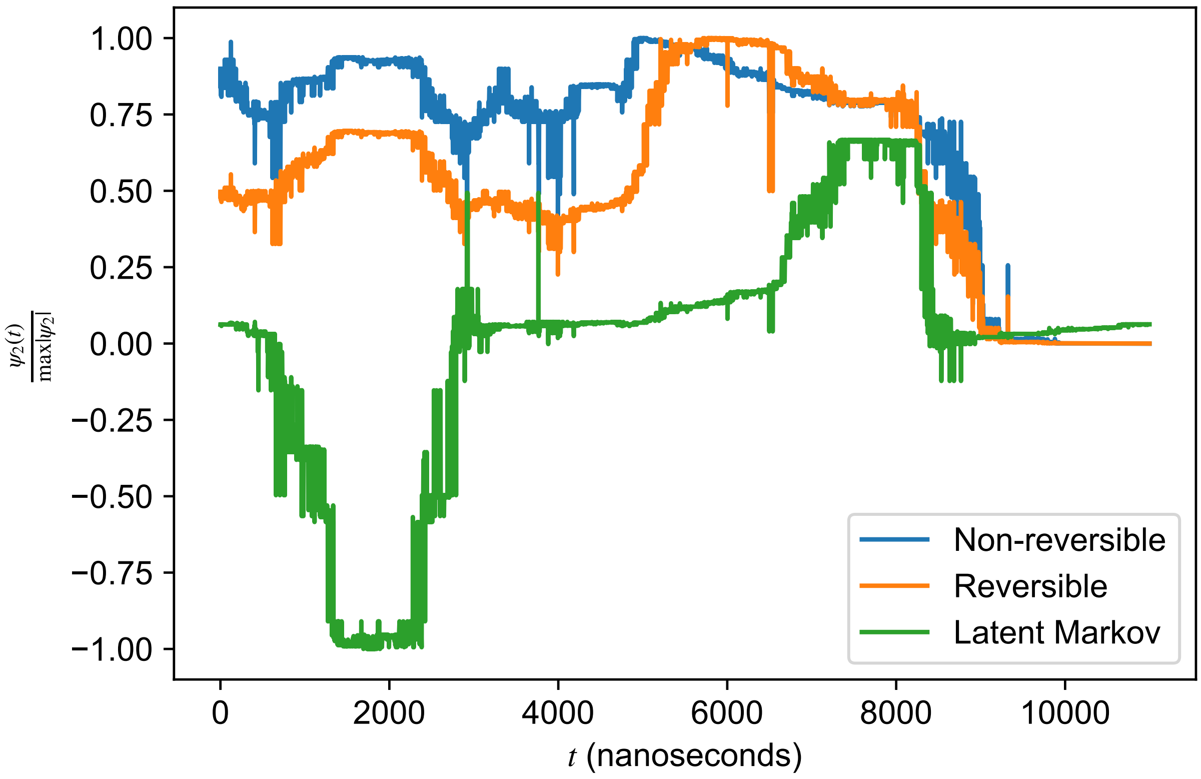

Figure S2: Comparisons of the α-synuclein trajectories projected onto ψ_2, second invariant measure normalized by first invariant measure. Estimators MSM (reversible, non-reversible) and Latent Markov model. Lag-time 21 nanoseconds for all. Please also see the Fig. 3A and 3B from the main manuscript for the comparison of the corresponding model quality measures.
